# Nonlinear optical response of graphene oxide quantum dots fabricated from electrospun polyacrylonitrile fibers

**DOI:** 10.1016/j.heliyon.2023.e12986

**Published:** 2023-01-16

**Authors:** Celia L. Gomez, O. Zaca Morán, M. Rojas-López, C. Morán-Raya, P. Zaca-Morán

**Affiliations:** a Instituto de Ciencias, Benemérita Universidad Autónoma de Puebla, CP 72050 Puebla, Mexico; b Instituto Politécnico Nacional, Centro de Investigación en Biotecnología Aplicada, Ex-Hacienda de San Juan Molino, Km 1.5 de la Carretera Estatal Santa Inés, Tecuexcomac-Tepetitla, Tepetitla, CP 90700, Tlaxcala, Mexico

**Keywords:** Graphene oxide quantum dots, Optical fibers, Nonlinear optical response, Nonlinear susceptibility

## Abstract

The nonlinear optical response of graphene oxide quantum dots (GOQDs) fabricated by the carbonization and exfoliation of electrospun polyacrylonitrile (PAN) fibers is reported. Electrospun and carbonized fibers were characterized by SEM and XPS. SEM micrograph confirmed the formation of PAN fibers of 153.44 ± 6.44 nm, while by XPS the binding energies associated with sp^2^ and sp^3^ carbon hybridizations were found, after the carbonization process. On the other hand, the GOQDs obtained were characterized by photoluminescence (PL), UV–Vis, Raman spectroscopy, and High-Resolution Transmission Electron Microscopy (HRTEM). The GOQDs size of 10 nm was estimated by HRTEM. Raman spectroscopy showed the D and G bands associated with the sp^2^ and sp^3^ hybridizations of the GOQDs, by PL two energy values of 2.67 and 2.97 eV were calculated. The UV–Vis spectrum showed two absorption bands confirming the presence of GOQDs. The nonlinear characterization was carried out using the P-scan technique, previously photodepositing GOQDs onto an optical fiber, using a coherent radiation source at a wavelength of 1550 nm. The results obtained showed a saturable absorption behavior with a value of $\beta ={-2.474\times 10}^{-4}\;m/W$ and a nonlinear susceptibility of $\chi^{\left(3\right)}\approx {-7.749\times 10}^{-4}\;\left(esu\right)$. The results of this work showed that GOQDs obtained can be used for optical switching applications.

## 1 Introduction

Nowadays, the study of the optical properties of materials at a low dimensional scale has been of great importance for the development of photonic technology due to its applications in high-speed optical communications [[1], [2], [3], [4]], optical limiters [5,6], solar cells [[7], [8], [9], [10]], biosensors [[11], [12], [13]], among others. In particular, the quantum dots (QDs) present phenomena that have recently been studied in the optics area due to their smaller dimensions than 10 nm, as well as for their nonlinear optical responses that make them attractive as saturable absorber (SA) devices [14]. SAs are photonic devices that allow full transmission at low optical powers and maintain a saturation level at high powers, thus maintaining a constant value of transmitted power [[15], [16], [17], [18], [19], [20]].

Mamour et al. reported the study of the linear and nonlinear optical properties of GOQDs, synthesized by exfoliation of graphite oxide obtained by the modified Hummers' method [21]. The nonlinear optical properties of the GOQDs, deposited by the drop casting method as thin films, were obtained using the Z-scan technique with a duration pulse of 5 ns at 532 nm. The results of this work showed a behavior characteristic of a SA with a value for the nonlinear absorption coefficient of $\beta ={5\times 10}^{-14}$ m/W, using 30 μJ of laser energy.

On the other hand, Gao et al. reported the nonlinear optical response of QDs fabricated by bulk black phosphorous (BP) exfoliation with phytic acid [16]. The nonlinear characterization of the black phosphorus quantum dots (BPQDs) obtained was performed by the Z-scan technique using an Nd:YAG laser with pulses of 4 ns duration. Their results showed saturable absorption effects at 532 and 1064 nm with nonlinear absorption coefficients of −1.5 × 10^−10^ and −1.7 × 10^−12^ m/W, respectively, making them ideal as SAs for these wavelengths.

In addition, Xu et al. reported the fabrication of BPQDs by electrospinning technique using a composite of polymethylmethacrylate (PMMA)/BPQDs nanofibers [19]. The composite was characterized using the Z-scan technique with a femtosecond laser at a wavelength of 800 nm. The results showed that the composite has a nonlinear absorption coefficient of −0.41 × 10^−3^ cm/GW, which can be used as SA for BP-based optoelectronic devices. The disadvantage of the aforementioned QDs synthesis methods lies in the use of strong acids and non-environmentally friendly reagents, as well as long time for obtaining them.

In this work, we report the fabrication and characterization of the nonlinear optical response of GOQDs obtained from carbonized and exfoliated PAN electrospun fibers. This fabrication technique of GOQDs has the advantage of being non-polluting or using polluting chemicals, which makes it novel, simple, and easy compared to reported methods. The nonlinear characterization of the GOQDs was performed using the P-scan technique, previously photodepositing them on an optical fiber using a coherent radiation source at a wavelength of 1550 nm.

## 2 Methods

### 2.1 Fabrication of GOQDs

**Fig. 1:**
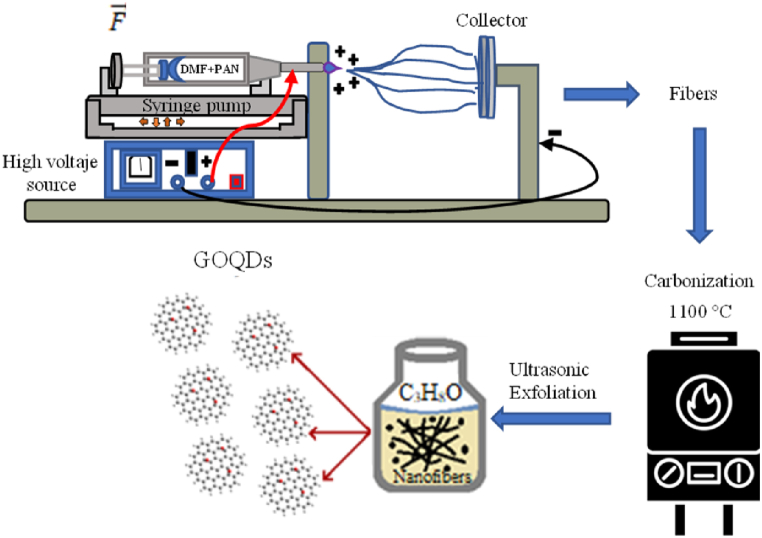
Schematic representation of the GOQDs fabrication process.

The fabrication of GOQDs was carried out by the electrospinning technique from PAN electrospun fibers previously reported by Zaca-Moran O. et al. [22]. The fibers were fabricated using an electrospinning system (Spellman High Voltage Dc Supply) at 15 kV, with a double syringe pump (Kd Scientific), a precursor flow rate of 0.5 ml/h, a linear force of 20 lb/min and a working distance (syringe-circular copper collector) of 15 cm. A polymeric solution was prepared by dissolving PAN in 10 wt % N–N-Dimethylformamide (DMF) and maintained under stirring for 12 h at room temperature. The PAN fiber fabrication was carried out by introducing the polymeric solution into the electrospinning system, during a collection time of 10 min at room temperature. The PAN fibers obtained were stabilized in an oven (Barnstead Thermolyne Furnace 1300) at 270 °C with a heating ramp of 5 °C/min for 30 min under atmospheric conditions. Subsequently, the PAN fibers were carbonized at 1100 °C with a heating ramp of 5 °C/min in a nitrogen atmosphere, to obtain graphite oxide fibers. Finally, the graphite oxide fibers were placed in an ultrasonic bath (Branson 3800) at 40 kHz in 2-Propanol (C_3_H_8_O) for 90 min to perform the exfoliation process and eventually obtain the GOQDs colloidal solution with a concentration of 2.3 mg/ml. The schematic representation of the GOQDs fabrication process is shown in Fig. 1.

### 2.2 Characterizations of PAN fibers and GOQDs

The surface morphology of the PAN fibers was characterized in a scanning electron microscope (SEM, TESCAN VEGA TS 5136SB) using high vacuum mode. The chemical analysis of carbonized fibers was carried out by X-ray Photoelectron Spectroscopy (XPS, Thermo Scientific K-Alpha) under ultra-high vacuum conditions (1 × 10^−9^ Torr) and a monochromatic X-ray source AlKα_1_ (hν = 1486.6 eV). The high-resolution spectrum was adjusted to determine the composition of the chemical elements that make up the sample. It is important to mention that the energy of all elements was calibrated using the C–C bond of C 1s (284.8 eV) as reference. The optical absorption spectrum of the GOQDs was obtained at room temperature using a UV–Vis scanning spectrophotometer (Thermo Scientific). The GOQDs Raman spectrum was recorded using a Thermo Scientific brand DXR Smart Raman spectrometer, using a 780 nm laser excitation source with 12 mW output power and a CCD detector thermoelectrically cooled; an operating range from 50 to 3350 cm^−1^ was used. The PL spectra were measured at ambient conditions by a spectrofluorophotometer using a He–Cd (Omnichrome-Series 56) laser emitting at 325 nm with an optical excitation power of ∼15 mW. To confirm the GOQDs morphology and estimate the GOQDs size HRTEM was performed using a JEOL JEM200 of 80–200 kV microscope with a CCD camera in real time to record the images obtained. To analyze the HRTEM micrographs, Gatan Digital Micrograph software was used.

### 2.3 Nonlinear optical response of the GOQDs

**Fig. 2:**
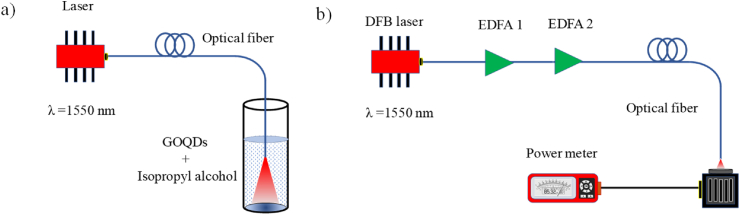
Experimental setup for the process of a) Photodeposition of the GOQDs, b) Characterization of the nonlinear optical response of the GOQDs.

The GOQDs photodeposition on the optical fiber was performed using the technique reported by Ortega-Mendoza et al. [23]. The technique consists of removing the plastic coating from a single-mode optical fiber (SMF-28) using a fiber stripper, followed by a transverse cut to have a uniformly cut fiber at its core. Subsequently, the fiber is immersed in a colloidal solution of GOQDs and an infrared laser radiation with emission at 1550 nm is passed until a loss of 1.2 dB is obtained at the end of the fiber, as shown in Fig. 2a. The photodeposition mechanism is due to the selective manipulation of nanoparticles by means of a coherent radiation source. A convective flow is induced by the radiation source at 1550 nm which interacts with the forces of absorption and scattering within the cone of radiation generated within the solution. The result is the trapping and selective photodeposition of the GOQDs on the core of the optical fiber [23].

The experimental setup for the characterization of the nonlinear optical response of the GOQDs photodeposited on the core of the optical fiber was performed using the P-scan technique reported by Zaca-Morán et al. [24]. The experimental setup consists of a Distributed Feedback (DFB) Laser with emission at 1550 nm coupled to two amplification stages, EDFA1 and EDFA2, by means of erbium-doped fibers as shown in Fig. 2b. The temporal width and frequency of the pulses used were 20 nm and 2 kHz, respectively; under these conditions, the system provided a maximum output irradiance of 400 MW/cm^2^, which was measured at the fiber output using a spherical integration photodetector (S145C, Thorlabs) and a power meter (PM100D, Thorlabs).

The nonlinear optical response of the photodeposited GOQDs on the optical fiber was modeled using the following equation [24]:

(1) $$\beta \left(I\right)=\frac{\beta }{1+I/I_{sat}}$$

where β(I) is the nonlinear absorption coefficient, β is the saturable absorption coefficient and $I_{sat}$ is the saturation intensity, defined as the intensity of the laser at which β drops to 50% of its initial value. Nonlinear absorption is a nonlinear optical effect in which the absorption coefficient depends on excitation intensity and the saturable absorption is a property of materials where the absorption of light decreases with increasing light intensity [15,16,24].

Using Eq. (1) the imaginary part of the susceptibility was calculated by $Im\;\chi^{\left(3\right)}=\frac{\lambda \varepsilon_{0}n_{0}^{2}c\beta }{4\pi }$ where $n_{0}$ is the linear refractive index of graphene ($n_{0}\approx 3.258$ for graphene [25]), $\varepsilon_{0}$ is the permittivity of free space (8.85 × 10^−12^ F/m), and *c* is the speed of light in vacuum [24]. This last expression gives units of m^2^/V^2^, so it was changed to susceptibility units (esu) using the conversion 1 m^2^/V^2^ = 9 × 10^8^ (esu).

## 3 Results

**Fig. 3:**
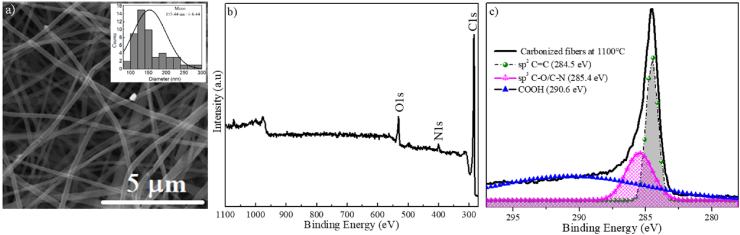
a) SEM micrograph of carbonized PAN fibers. Inset: Histogram of carbonized PAN fibers diameter distributions, b) XPS survey spectrum of carbonized PAN fibers, and c) Deconvolution of the high-resolution spectrum corresponding to the carbon peak in carbonized PAN fibers.

Fig. 3a presents SEM micrograph of PAN fibers uniformly distributed and randomly oriented, with homogeneous morphology, without defects such as rosaries, ribbons like or others [26]. The histogram of carbonized PAN fibers diameter distributions with an average of 153.44 ± 6.44 nm is shown in the inset of Fig. 3a. The XPS survey spectrum of carbonized PAN fibers showed bonds associated with oxygen (O1s), nitrogen (N1s), and carbon (C1s) elements (Fig. 3b). The deconvolution of the peak corresponding to carbon (C1s) of the high-resolution XPS spectrum (Fig. 3c) was performed, identifying the sp^2^ and sp^3^ hybridizations, as well as the presence of functional groups associated with graphite oxide. The energy values calculated for the bonds C–C and Cce:glyph name="dbnd"/>C were 284.5 eV associated with the sp^2^ hybridization and 285.4 eV corresponding to the C–H, C–O, and C–N bonds of the sp^3^ hybridizations; in addition to an energy of 290.6 eV related to the C–O and O–Cce:glyph name="dbnd"/>O bonds of the carboxyl functional group (COOH) [[27], [28], [29]].

**Fig. 4:**
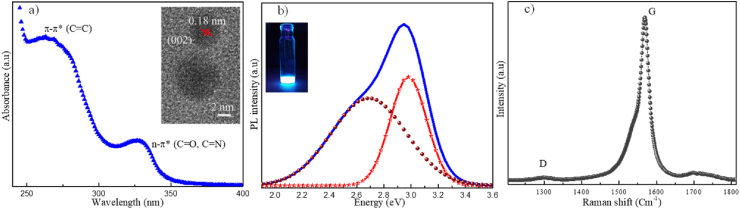
(a) UV–vis spectrum of GOQDs. Inset: HRTEM micrograph of GOQDs, (b) PL spectrum of GOQDs. Inset: Photograph of the characteristic blue emission of the GOQDs, and (c) Raman spectrum of GOQDs. (For interpretation of the references to colour in this figure legend, the reader is referred to the Web version of this article.)

Fig. 4a shows the UV–Vis spectrum of GOQDs with an absorption band around 265 nm, assigned to the electronic transitions π → π* of the π bonds of the Cce:glyph name="dbnd"/>C aromatic ring in its structure, as well as an additional band around 325 nm related to the n → π* transitions of Cce:glyph name="dbnd"/>O bonds. According to the reported by Ostovari et al. [30], these bands are characteristic of GOQDs. The inset in Fig. 4a shows the HRTEM micrograph of the GOQDs with sizes ∼10 nm with a lattice parameter of 0.18 nm associated with (002) plane of graphene oxide [31]. Fig. 4b shows the photoluminescence spectrum of the GOQDs where two emission bands are observed at 2.67 and 2.97 eV, respectively. These emissions are associated with the confinement of electrons in the GOQDs and the size particles [31]. The inset in Fig. 4b shows the photograph of the GOQDs-isopropyl alcohol colloidal solution irradiated with a UV lamp, where the characteristic emission of the GOQDs is observed in the blue region. Fig. 4c shows the characteristic Raman spectrum of the GOQDs, observing two predominant bands, one at 1298 cm^−1^ called the D band associated with second-order resonances and another at 1566 cm^−1^ known as G band own to C–C bonds. The G band is predominant which indicates that the GOQDs obtained present fewer defects in the network, these bands are similarly related to sp^2^ and sp^3^ hybridizations [32]. It is important to mention that, according to the reported literature on the nonlinear properties of graphene-based materials, their nonlinear absorption properties are directly associated with sp^2^ and sp^3^ hybridizations of GOQDs due to carbon atoms, as well as to conjugated structures and π−π* bonds [33,34].

**Fig. 5:**
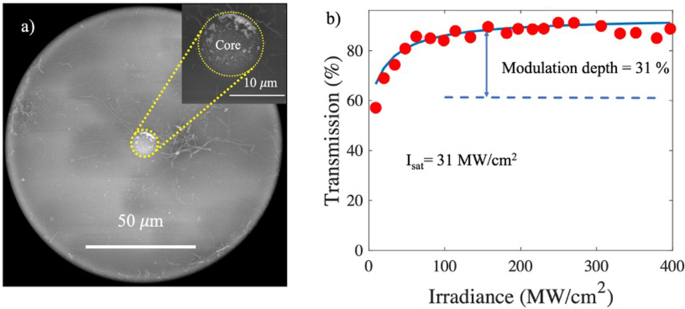
(a) SEM micrograph of the cross-section of the optical fiber with the photodeposited GOQDs. Inset: Close-up of the optical fiber core, and (b) nonlinear optical response of the GOQDs photodeposited on the core of an optical fiber.

Fig. 5a shows the SEM micrograph of the cross section of a single mode optical fiber with the GOQDs photodeposited on its core, where the core of the optical fiber (dotted circle) can be seen surrounded by a small number of fibers PAN unevenly distributed on the surface of the cladding. The inset of Fig. 5a shows a close-up of the core where the surface covered with GOQDs can be seen. It is important to mention that the materials surrounding the core of the optical fiber do not interact with the propagated electromagnetic field; therefore, they do not contribute to the nonlinear characterization of the GOQDs.

Fig. 5b shows the results obtained from GOQDs photodeposited on the core of an optical fiber under the influence of an intense radiation field. A linear transmission is observed, which increases for intensities below 50 MW/cm^2^ and saturation for higher intensity values, thus showing a saturable absorption behavior with a value of $\beta ={-2.474\times 10}^{-4}\;m/W$ and a nonlinear susceptibility of $\chi^{\left(3\right)}\approx {-7.749\times 10}^{-4}\;esu$, respectively. This shows that the fabricated GOQDs have the property of absorbing radiation at high input optical intensity, which is a typical behavior of devices with nonlinear optical properties. Measurements were repeated several times under the same conditions to ensure the reproducibility of the results, which means that there are no heating effects that modify the structural or morphological properties of the GOQDs.

According to the obtained results, it was observed that when the GOQDs are under the influence of intense radiation fields, these show a typical behavior of a SA. Here, the electrons are excited at the same time, until the conduction band is filled (Pauli blocking) preventing the absorption of more photons. Pauli blocking implies that while the electrons are being transmitted to the conduction band, it will cause a saturation effect [35,36].

## 4 Conclusions

In the present work, the fabrication of GOQDs with ∼10 nm of diameter through the carbonization and exfoliation of electrospun PAN fibers was reported. The sp^2^ and sp^3^ hybridizations of carbon were confirmed by XPS and the π−π* and n−π transitions of the GOQDs were identified by UV–Vis spectroscopy, these structural properties of the GOQDs are associated with materials with nonlinear properties. Raman and PL results showed the presence of sp^2^ and sp^3^ hybridizations of the GOQDs which are associated with their nonlinear properties. The GOQDs were photodeposited on the core of an optical fiber and the SEM micrographs of the optical fiber core confirmed their photodeposition. GOQDs photodeposited on the core of an optical fiber were characterized by an intense radiation source, showing a saturable absorption behavior characteristic of devices with nonlinear properties. The results obtained in this work demonstrate that GOQDs can be used to fabricate high-speed photonic devices.

## Author contribution statement

Celia L. Gomez, PhD: Performed the experiments; Analyzed and interpreted the data; Wrote the paper.

Orlando Zaca, PhD; Placido Zaca-Moran, PhD: Conceived and designed the experiments; Performed the experiments; Analyzed and interpreted the data; Wrote the paper.

Marlon Rojas, PhD; Carolina Morán, PhD: Analyzed and interpreted the data; Contributed reagents, materials, analysis tools or data; Wrote the paper.

## Funding statement

This work was supported by CONACyT-México through the grant No. BP-PAIM-20220906151734769-3682852, VIEP-BUAP under grant No. 365/2022 as well as 10.13039/501100003069National Polytechnic Institute under project 20220613.

## Data availability statement

Data will be made available on request.

## Declaration of interest's statement

The authors declare that they have no known competing financial interests or personal relationships that could have appeared to influence the work reported in this paper.
